# Two novel variations in *LRP2* cause Donnai-Barrow syndrome in a Chinese family with severe early-onset high myopia

**DOI:** 10.3389/fgene.2023.1107347

**Published:** 2023-01-27

**Authors:** Shiqin Yuan, Xiaoyu Huang, Shuang Zhang, Shangying Yang, Xue Rui, Xiaolong Qi, Xuhui Wang, Yali Zheng, Weining Rong, Xunlun Sheng

**Affiliations:** ^1^ Ningxia Eye Hospital, People’s Hospital of Ningxia Hui Autonomous Region, Third Clinical Medical College of Ningxia Medical University, Yinchuan, China; ^2^ Clinical Medical College, Ningxia Medical University, Yinchuan, China; ^3^ Gansu Aier Ophthalmology and Optometry Hospital, Lanzhou, China; ^4^ Department of Kidney Internal Medicine, People’s Hospital of Ningxia Hui Autonomous Region, Third Clinical Medical College of Ningxia Medical University, Yinchuan, China

**Keywords:** Donnai-Barrow syndrome, early-onset high myopia, compound heterozygous variation, clinical features, genetic assessment

## Abstract

Donnai-Barrow syndrome (DBS) is a rare autosomal recessive disorder caused by mutation in the low density lipoprotein receptor-related protein 2 gene (*LRP2*). Defects in this protein may lead to clinical multiple organ malformations by affecting the development of organs such as the nervous system, eyes, ears, and kidneys. Although some variations on *LRP2* have been found to be associated with DBS, early diagnosis and prevention of patients with atypical DBS remains a challenge for many physicians because of their clinical heterogeneity. The objective of this study is to explore the association between the clinical presentation and the genotype of a DBS patient who was initially diagnosed with early-onset high myopia (eoHM) from a healthy Chinese family. To this end, we tested the patient of this family *via* whole exome sequencing and further verified the results among other family members by Sanger sequencing. Comprehensive ophthalmic tests as well as other systemic examinations were also performed on participants with various genotypes. Genetic assessment revealed that two novel variations in *LRP2*, a *de novo* missense variation (c.9032G>A; p.Arg3011Lys) and a novel splicing variation (c.2909-2A>T) inherited from the father, were both carried by the proband in this family, and they are strongly associated with the typical clinical features of DBS patients. Therefore, in this paper we are the first to report two novel compound heterozygous variations in *LPR2* causing DBS. Our study extends the genotypic spectrums for *LPR2*-DBS and better assists physicians in predicting, diagnosing, and conducting gene therapy for DBS.

## Introduction

DBS is an extremely rare and complex disorder with wide phenotypic variability. It is characterized by major malformations including agenesis of the corpus callosum, congenital diaphragmatic hernia, facial dysmorphology, ocular anomalies, sensorineural hearing loss, developmental delay, and low molecular weight proteinuria (retinal-binding protein and vitamin D-binding protein are mainly elevated in urine). Only about 62 individuals worldwide to date with distinct DBS features have been reported in the medical literatures and there are no other population-based incidences or prevalence data (Avunduk et al., 2000; Chassaing et al., 2003; Kantarci and Donahoe, 2007; Patel et al., 2007; Kantarci et al., 2008; Shaheen et al., 2010; Bruce et al., 2011; Chinta et al., 2011; Roane et al., 2012; Storm et al., 2013; Schrauwen et al., 2014; Dachy et al., 2015; Khalifa et al., 2015; Anglani et al., 2018; Khan and Ghazi, 2018; Longoni et al., 2018; Canut et al., 2020; Flemming et al., 2020; Ozdemir et al., 2020; Aksenova et al., 2021; Dumitrescu et al., 2021; Higham et al., 2021; Robinson et al., 2021; Sait et al., 2021; Alyousef et al., 2022). An increasing number of studies (Kantarci et al., 2007; Kantarci et al., 2008; Shaheen et al., 2010; Storm et al., 2013; Schrauwen et al., 2014; Khalifa et al., 2015; Anglani et al., 2018; Flemming et al., 2020; Ozdemir et al., 2020; Aksenova et al., 2021; Higham et al., 2021) have found that DBS is caused by variations in the *LRP2* gene, which locates on chromosome 2q24-31 and encodes the megalin protein, a multiligand endocytic receptor that plays an important role in retinal and renal tissues by mediating endocytic uptake and clearance of sonic hedgehog in the retinal margin and by mediating renal uptake of the antiapoptotic protein survivin (Jobst-Schwan et al., 2013; Christ et al., 2015). As recorded by the human genetic mutation database by April of 2021, although 94 *LRP2* variations have been found to be associated with many diseases, such as autism, cancer, obesity, and congenital heart defect, only few of them have been linked to DBS. Therefore, molecular diagnosis as a reliable tool for diagnosis when the pathogenic variation is known may be limited by those unknown variations in this disease.

Studies in mammals demonstrate LRP2 expression in the ciliary epithelia, retinal pigment epithelium layer, and retinal neurosensory layer of eye, which is critical for normal ocular function (Zheng et al., 1994; Assémat et al., 2005). High myopia is divided into early-onset high myopia (eoHM) and late-onset high myopia (loHM) according to age of onset. eoHM is defined as refractive error ≤ -6.0 D or an eye axis length>26 mm and occurs at preschool age (less than 7 years old) (Morgan and Rose, 2005). High myopia and hypertelorism are prevalent major features of DBS, typically with down-slanting palpebral fissures. However, fewer studies have analyzed the phenotype of DBS-eoHM patient with *LRP2* variation. In this paper, we describe one DBS patient with eoHM carrying two novel *LRP2* variations from a healthy Chinese family and discuss the phenotypic characteristics of individuals with different genotypes, providing reliable molecular diagnosis for the DBS and prenatal screening aid to similar families to have healthier offspring.

## Materials and methods

### Clinical observations and analysis

This study was undertaken according to the Declaration of Helsinki and was approved and reviewed by the Ethics Committee on Human Research at People Hospital of Ningxia Hui Autonomous Region. Informed consent was obtained from all participants including parents and children.

All individuals in this family were recruited for both clinical and genetic tests. Comprehensive ophthalmic examinations and other systemic examinations were performed on subjects, including best-corrected visual acuity (BCVA), axial length (AL), corneal curvature (CC), anterior chamber depth (ACD), color fundus photography, visual fields (VF), full-field electroretinography (ffERG), optical coherence tomography (OCT), ultrasound of eyes and uterus, and 24-h proteinuria examinations.

BCVA was recorded by snellen visual acuity chart and optometry instrument (VT-10, TOPCON, Japan), respectively. AL, CC, and ACD were recorded by IOL marst optical biometry (IOL Marster 500, Carl Zeiss Meditec AG). Images of color fundus photography were captured from fundus photography analyzer (TRC-NW300, TOPCON, Japan). The visual field testing was performed with humphrey field analyzer (750i Carl Zeiss Meditec, United States). ffERG was performed using corneal ERG jet contact lens electrodes (RetiPort ERG system; Roland Consult, Wiesbaden, Germany), according to the international society of clinical electrophysiology of vision standards (Marmor et al., 2009; McCulloch et al., 2015). Enhanced depth imaging (EDI) of cirrus high definition optical coherence tomography (HD-OCT4000, Carl Zeiss Meditec, United States) was performed on the foveal center of macular cube. The pictures of ultrasound of eyes and uterus were captured by ultrasound diagnostic system (Mylab75, EIZO NANAO Corp.). Detect 24-h proteinuria by a urine total protein (CSF/UP) assay kit (Gcell, Beijing Strong Biotechnologies, Inc.) according to the manufacturer’s instructions.

Peripheral venous blood samples (3 mL) were collected from all individuals of the family for genomic DNA isolation. DNA isolation was performed based on the manufacturer’s instructions of TIANamp blood DNA kit (#DP348-03, TIANGEN biotech (Beijing) co., ltd).

### Sequencing analysis

Proband’s whole-exome library was captured by using xGen Exome Research Panel v1.0 of IDT (Integrated DNA Technologies, Inc., United States). Then the product was quantified after purification by magnetic beads. Paired-end sequencing was performed on an Illumina (San Diego, CA) sequencing platform using PE150 mode. Sequencing data analysis first need to remove the reads that did not meet the quality control requirements in the original sequencing data, and then use BWA (Burrows–Wheeler Aligner) software to align with the hg19 version of the human genome reference sequence provided by UCSC (https://genome.ucsc.edu/), and finally find out the variants by GATK v3.70 (Genome Analysis Toolkit). The pathogenicity classification of variants refers to the genetic variant classification standards and guidelines of American College of Medical Genetics and Genomics (ACMG). Several publicly available servers for bioinformatic prediction tools such as MutationTaster (http://www.mutationtaster.org/), FATHMM-MKL (http://fathmm.biocompute.org.uk/fathmmMKL.htm), PolyPhen-2 (http://genetics.bwh.harvard.edu/pph2/), dbscSNV (http://asia.ensembl.org/info/docs/tools/vep/script/vep_plugins.html#plugins_existing), and SpliceAI (https://github.com/Illumina/SpliceAI) were used to predict the effect of two novel variations of the DBS-associated pathogenic gene (*LRP2*) on its protein function. Then the variations of the studied gene were verified in proband’s parents and her siblings through Sanger sequencing.

Conservational analysis was first performed with UNIPROT knowledgebase (https://www.uniprot.org) to acquire the amino acid sequence of LRP2 from different species. The subsequent results were aligned and analyzed by alignment tool, Multalin (http://sacs.ucsf.edu/cgi-bin/multalin.py). The minor change of the amino acid at position 3011 between these sequences will show that the amino acid of LRP2 at this position is highly conserved among species and the p.Arg3011Lys in LRP2 is more likely to affect the structure and function of the related protein.

### Structural analysis

Protein modeling of the wild type and mutant LRP2 were constructed with SWISS-MODEL online server (https://swissmodel.expasy.org/interactive), and then displayed with PyMol software (https://pymol.org/2/).

## Results

### Medical history and external characteristics of patient in this family

**FIGURE 1:**
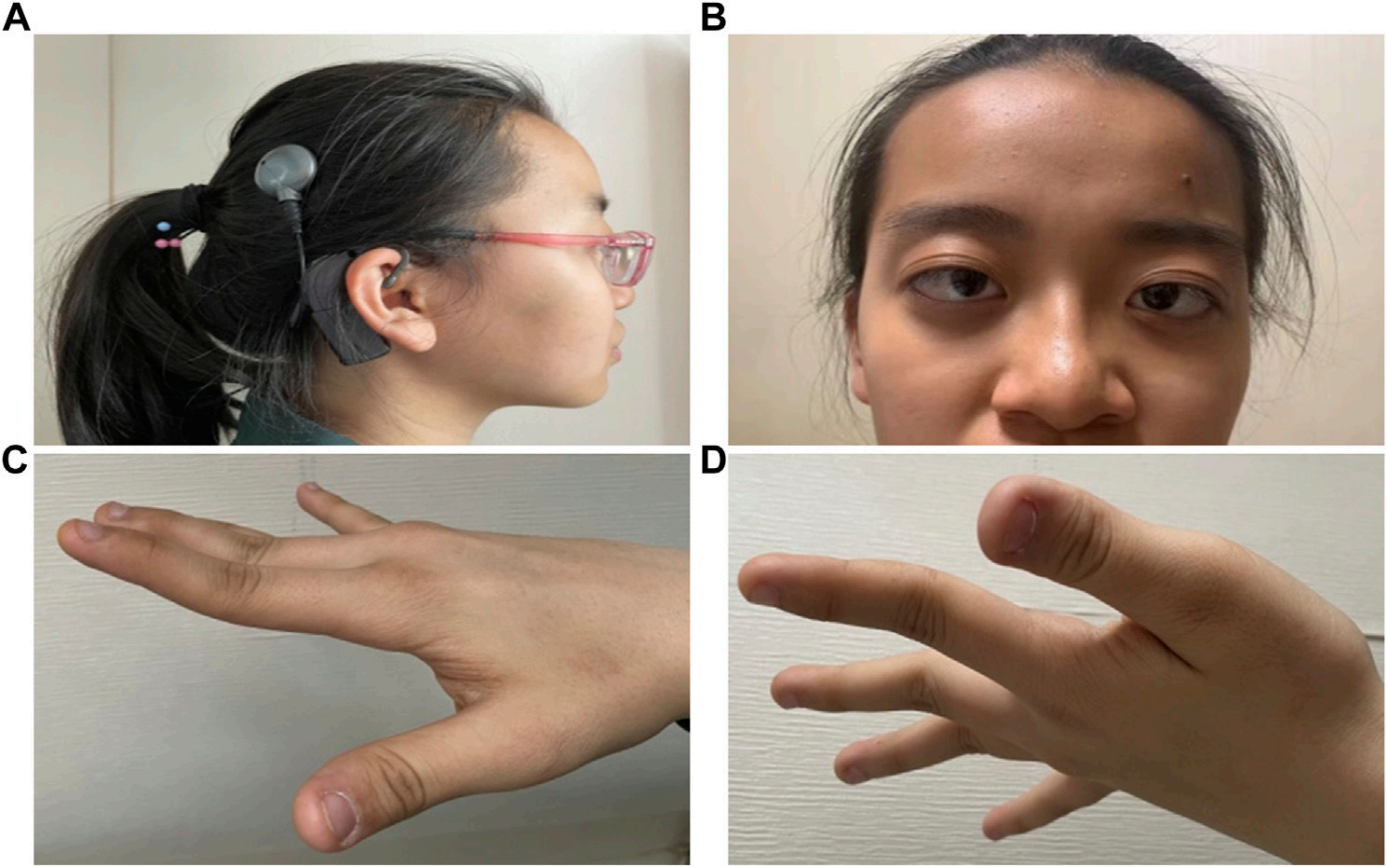
External photographs of DBS patient with early-onset high myopia. Patient is characterized by **(A)** Carrying hearing-aid as well as cochlear implant. **(B)** Ocular hypertelorism, exophthalmos, broad and high forehead, receding hairline, depressed nasal bridge, short nose with broad tip, and esotropia. **(C, D)** Hypermobility of the hand joints.

The proband (II:2) is a 14-year-old girl from a healthy unrelated couple (I:1, I:2). She has three healthy siblings (II:1, II:3, and II:4) of ages 13, 8, and 4 respectively. But she was born calcium deficient and had been breast-fed for only 2 months. At 2 months of age, she was detected with proteinuria. At 2 years of age, she was diagnosed with high myopia and sensorineural hearing loss and started wearing hearing aids (Figure 1A). For the following 3 years, she received the cognitive rehabilitation training. She did not learn to speak “mum” until 3 years old and continued to speak in a basic and unclear manner. At 9 years of age, she was performed ears magnetic resonance imaging (MRI) scanning and then was implanted cochlear in the right ear. At 13 years of age, she had her first period. At the time of the study, she is studying in a special school. She gets decent grades, participates adequately in sports she likes, and even takes bus home by herself.

A recent ophthalmologic examination of the patient II:2 in our hospital revealed that except for dysplasia in the ears and renal, the patient also had features of ocular hypertelorism, exophthalmos broad and high forehead, receding hairline, depressed nasal bridge, short nose with broad tip, and esotropia (Figure 1B). The fingers of both hands were overstretched (Figures 1C,D). Therefore, its clinical complications are serious enough to warrant genetic analysis.

### Two novel variations of *LRP2* are probably pathogenic factors of patient in our study

**FIGURE 2:**
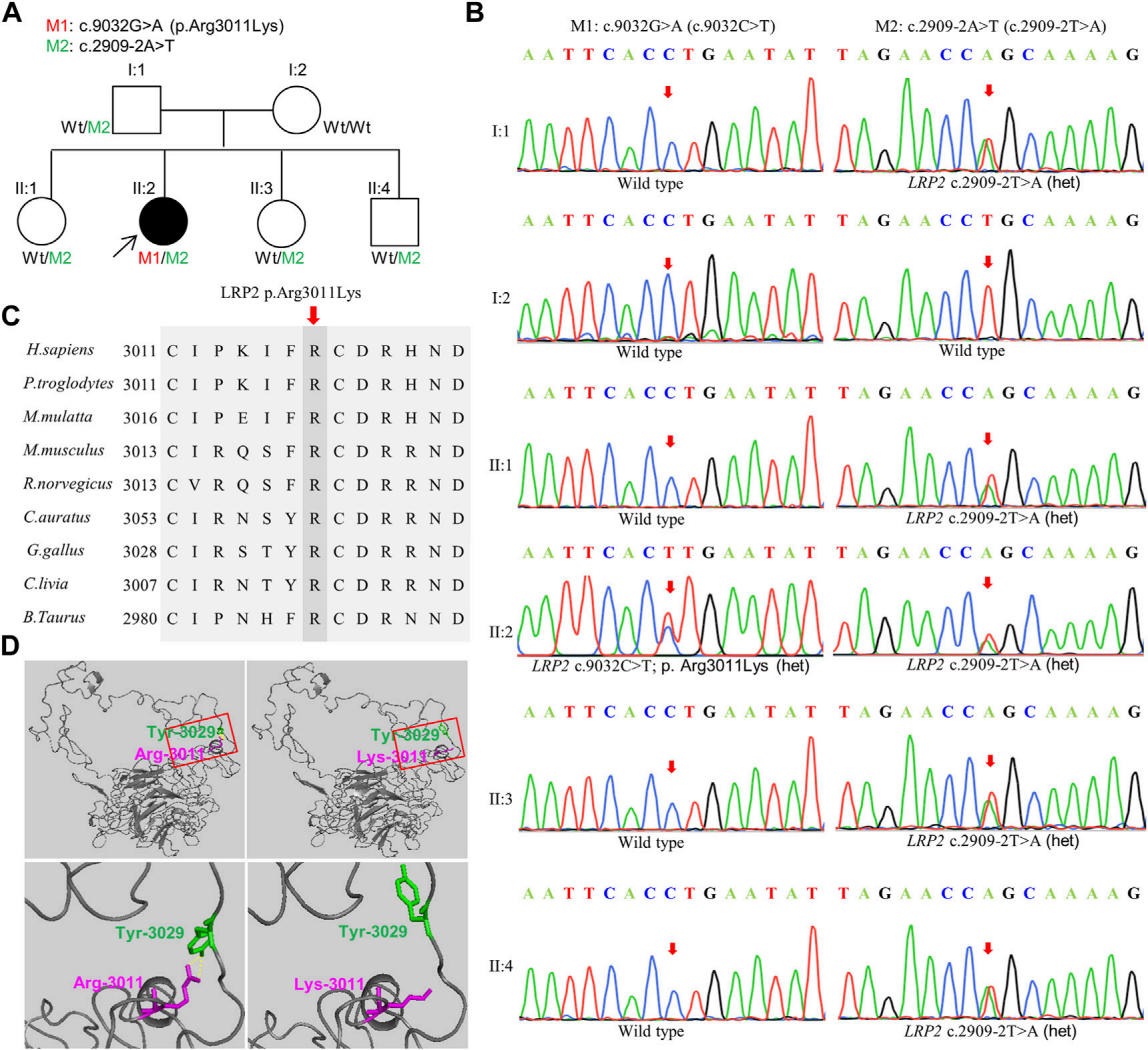
Validation of the variation. **(A)** Pedigree information. Filled black symbol represents the affected member. Two variations are M1: c.9032G>A (p.Arg3011Lys) and M2: c.2909-2A>T, Wt indicates Wild type. Proband is indicated by arrow. **(B)** Complementary sequence chromatograms of identified sites for all family members. **(C)** Orthologous protein sequence alignment of LRP2 from human (H. sapiens), chimpanzees (P. troglodytes), rhesus macaque (M.mulatta), mouse (M. musculus), rats (R. norvegicus), Goldfish (C.auratus), Chicken (G. gallus), Rock dove (C.livia), and cows (B. taurus). Conserved residues are shaded. **(D)** Structural analysis of the wild type and mutant LRP2 protein. Hydrogen bonds between residue 3011 and Tyr3029 were eliminated due to the substitution from arginine to lysine. Overall structure of human LRP2 and effect of the M1: c.9032G>A (p.Arg3011Lys) (top), Squares within solid red lines are cropped for magnification (bottom). The yellow dashed line indicates hydrogen bonds.

To identify the cause of the disease in this study, we performed the whole exome sequencing (WES) of the patient II:2 and found that two novel variations in *LRP2*, Mutation 1: c.9032G>A (p.Arg3011Lys) (M1), and Mutation 2: c.2909-2A>T (M2), were both carried by this patient. Further verification in other family members (I:1, I:2, II:1, II:3, and II:4) by Sanger sequencing showed that c.9032G>A (p.Arg3011Lys) was *de novo* missense variation and c.2909-2A>T was new splicing variation inherited from the father (I:1) (Figures 2A,B). Both variations were then performed *in silico* analysis by the prediction programs of FATHMM-MKL, Polyphen2 (HDIV/HVAR), MutationTaster, dbscSNV (Ada_2 score and Rf_score), and SpliceAI, all showing pathogenicity (Table 1). Besides, the arginine at position 3011 of LRP2 (the accession number of NCBI reference sequence of LRP2 protein is NP_004516.2) was highly conserved among different species by proteomic conservation analysis (Figure 2C), indicating that the missense variation at this site is more likely to affect the structure and function of LRP2 protein. What’s more, structural analysis of the wild type and variant LRP2 protein revealed that hydrogen bonds between residue Arg3011 and Tyr3029 were eliminated due to the substitution from arginine to lysine (Figure 2D), indicating that the novel *de novo* missense variation (c.9032G>A; p.Arg3011Lys) probably impact on the tertiary structure of LRP2 protein. Taken together, these two new variations in *LRP2* are likely to cause disease by affecting the structure and function of LRP2 protein.

**TABLE 1:** The effects of LRP2 variations on their protein function by *in silico* analysis.

Software	Variants	Score	Predicted signal
MutationTaster	c9032G>A (p.Arg3011Lys)	1	Disease_causing
fathmm-MKL	c9032G>A (p.Arg3011Lys)	0.99505	Damaging
Polyphen2 (HDIV/HVAR)	c9032G>A (p.Arg3011Lys)	0.999/0.996	Damaging
DbscSNV (ada_score)	c.2909-2A>T	0.999988	Probably affect splicing
DbscSNV (rf_score)	c.2909-2A>T	0.932	Probably affect splicing
SpliceAI	c.2909-2A>T	0.95	Accepter Loss (DS_AL)

### The patient with compound heterozygous variations of *LRP2* in this family presents obvious clinical phenotype of DBS

**FIGURE 3:**
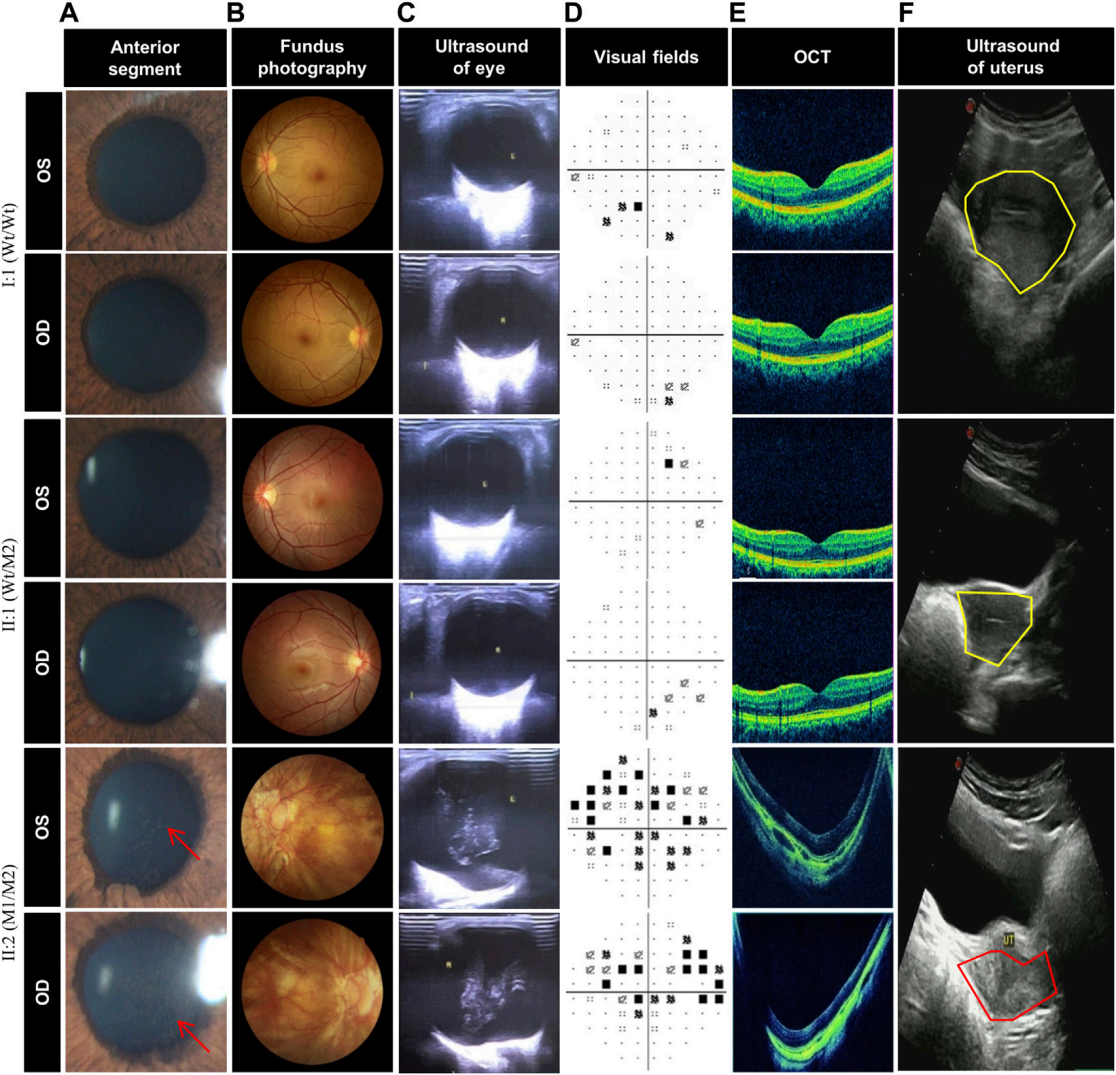
Clinical photograph of DBS patient with early-onset high myopia patient and unaffected members. **(A)** Anterior segment photography of the individuals I:2, II:1, and II:2, showing persistent pupillary membrane (red arrow) in II:2 but normal anterior segment in I:1 and II:1. **(B)** Fundus photography shows severe high myopic retinopathy in II:2 but no change in I:1 and II:1. **(C)** Ocular color duplex ultrasonography of the individuals I:2, II:1, and II:2, showing bilateral posterior staphylomata and vitreous opacities in both eyes of II:2 while no change in I:1 and II:1. **(D)** Visual field of II:2 shows small central scotoma and multiple paracentral scotoma in both eyes, but only very small paracentral scotoma were detected in both eyes of I:2 and II:1. **(E)** Optical coherence tomography of the individuals I:2, II:1, and II:2, showing fovea macula disappeared in II:2 but normal fovea macula in I:1 and II:1. **(F)** Uterine color ultrasound of the individuals I:2, II:1, and II:2, showing dicornal uterus (red coil) in II:2, but the uteruses (yellow coil) of I:1 and II:1 are normal, and the uterus of II:1 is larger than that of I:2 and II:1.

To explore whether the novel variations of *LRP2* cause distinguishing phenotypes in our patient, we selected subjects with three genotypes (Wt/Wt, Wt/M2, and M1/M2) in this family, all female (I:2, II:1, and II:2) (Figure 2A). Compared with the normal clinical manifestations of the mother (I:2) with wild type (Wt/Wt) and the younger sister (II:1) with heterozygous variation (Wt/M2), we found that the patient II:2 with compound heterozygous variations (M1/M2) in our study exhibited more severe ocular abnormalities, such as longer axial length (OD:37.58 mm, OS:37.80 mm), nystagmus, severe decline scotopic as well as photopic (Table 2), persistent pupillary membrane (Figure 3A), severe high myopic retinopathy in fundus (Figure 3B), bilateral posterior staphylomata and vitreous opacities (Figure 3C), small central scotoma and multiple paracentral scotoma in both eyes’ visual field (Figure 3D), and fovea macula disappeared (Figure 3E), and also showed dicornal uterus and proteinuria (2434.95 mg/24 h) (Figure 3F; Supplementary Table S1). It indicates that the patient II:2 in this family carries an autosomal recessive disorder caused by *LRP2* variations, and its main clinical features, including high myopia and dysplasia in ears, uterus, and renal, are consistent with that of DBS.

**TABLE 2:** Ophthalmological examination of DBS patient with early-onset high myopia and unaffected members.

Samples (genotype)	Sex	Age	Eye	BCVA	Refraction	AL (mm)	CC(D)	ACD	ffERG	Others
					(SE, D)			(mm)		
I:2(Wt/Wt)	F	35	OD	20/20	−1.50	22.38	44.70/45.12	2.66	Normal	None
			OS	20/20	−2.25	22.47	44.47/44.70	2.77		
II:1(Wt/M2)	F	13	OD	20/20	−2.75	24.24	43.35/44.29	3.57	Normal	None
			OS	20/20	−2.50	23.96	43.60/44.76	3.53		
II:2(M1/M2)	F	14	OD	20/100	−28.00	37.58	39.94/41.26	2.27	Severe decline	Nystagmus
			OS	20/100	−28.00	37.80	40.37/42.35	2.46		

Wt:Wildtype, M1:c9032G>A(p.Arg3011Lys), M2:c.2909-2A>T, F:Female, OD:oculus dexter, OS:oculus sinister, BCVA:Best-Corrected Visual Acuity, SE:spherical equivalent, D:Diopter, AL:axial length, CC:corneal curvature, ACD:anterior chamber depth, mm:Millimeter, ffERG:full-field Electrophsiology.

## Discussion

The patient II:2 in this study has clinical features concordant with DBS and harbors two novel LRP2 variations, which are very likely to be the cause of this disease. Hitherto, only about 25 different *LRP2* variations associated with DBS have been documented (Kantarci et al., 2007; Kantarci et al., 2008; Shaheen et al., 2010; Storm et al., 2013; Schrauwen et al., 2014; Khalifa et al., 2015; Anglani et al., 2018; Flemming et al., 2020; Ozdemir et al., 2020; Aksenova et al., 2021; Higham et al., 2021). They showed that there was no variant hotspot of *LRP2* and no obvious genotype-phenotype correlations (Kantarci et al., 2007; Khalifa et al., 2015; Anglani et al., 2018). Notably, most of the variations studied to date are homozygous variation expected to lead to the absence of LRP2 synthesis, causing distinct phenotypes in DBS (Kantarci et al., 2007; Shaheen et al., 2010; Khalifa et al., 2015). Furthermore, LRP2 has not been reported to be expressed in fibroblasts and lymphoblasts, and accordingly, we were unable to detect the protein or mRNA of LRP2 in the blood of our DBS patient by immunoblotting or real-time PCR. However, in this study, we can predict that two novel compound heterozygous variations of *LRP2* carried by the patient II:2 probably affect the function of LRP2 protein primarily by impacting on its structure as well as synthesis, leading to different DBS phenotypes. As shown by the structural analysis of wild type and variant protein of LRP2, the hydrogen bonds between residue Arg3011 and Tyr3029 were eliminated due to the substitution from arginine to lysine (Figure 2D), indicating that the *de novo* missense variation (c.9032G>A; p.Arg3011Lys) of *LRP2* is likely to cause reduced protein stability by affecting the tertiary structure of the LRP 2 protein.

In addition, both variations in this study were also predicted to be deleterious through *in silico* analysis of mutationTaster, FATHMM-MKL, polyphen2 (HDIV/HVAR), dbscSNV, and SpliceAI (Table 1). The p.Arg3011Lys is a novel missense variation located in the extracellular domain of LRP2 and is conserved among species (Figure 2C). The large amino-terminal extracellular domain of LRP2 contains four clusters (Ⅰ-Ⅳ) of low-density lipoprotein receptor (LDL) type A repeats that constitute the ligand-binding regions (Russell et al., 1989; Fass et al., 1997). p.Arg3011Lys in this study might be impairing part of LRP2 protein’s function by reducing certain ligand-binding affinities located in the extracellular domain of LDL. *LRP2* has a total of 79 exons. Another new variation, c.2909-2A>T, is located at the 3′end of intron 20 of *LRP2* gene, close to the 5′flanking of *LRP2*. As other studies discussed, a transcript that contains premature translational-termination codon (PTC) closing to the 5′flanking of gene is easily recognized by mRNA quality monitoring mechanism, non-sense-mediated mRNA decay (NMD), and rapidly degraded, thus eliminating abnormal transcript. NMD often affects the clinical phenotype of monogenic inherited diseases by altering the disease type, severity, and inheritance mode (Holbrook et al., 2004; Conti and Izaurralde, 2005; Khajavi et al., 2006; Bhuvanagiri et al., 2010; Pomares et al., 2012). Therefore, according to the results of *in silico* analysis (Table 1), the splicing variation in this family also probably causes the production of PTC, which is generally harmful to organisms, due to certain intron retention or exon hopping of *LRP2*, and subsequently results in the loss of LRP2 functionality.

Previously reported DBS, also known as facio-oculo-acoustico-renal syndrome, is a rare autosomal recessive and multisystem condition involving craniofacial abnormalities, ocular abnormalities, sensorineural hearing loss, nephropathy, agenesis of the corpus callosum, and developmental delay. Comparing the system features about DBS between the literature and the patient presented in this study, we found that most DBS patients presented with high myopia, sensorineural hearing loss, proteinuria, and hypertelorism (Supplementary Table S1). Similarly, the patient II:2 in our study also had obvious dysplasia in the eyes, ears, and kidneys, especially the clinical phenotypes of bicornuate uterus and joint hypermobility of patient II:2 were rarely addressed in prior DBS patients (Supplementary Table S1). But the features of other organs of the patient in here were relatively mild or normal. In particular, the patient II:2 had not been detected with omphalocele or abnormality in cerebral parenchyma (Supplementary Table S1). Hence, familial variability, which is a feature of DBS is probably explained by the genetic background of each patient and, possibly, by some random factors.

*LRP2* is primary cilia related gene associated with cilia transduced cell signaling pathway which is crucial to the formation, normal development and maintenance of visual, auditory and olfactory organs, central nervous system, respiratory system, gonads, liver, kidney and other tissue cells. Its protein function loss can lead to a range of clinical phenotypes including retinal dystrophy, hearing loss, neurodevelopmental defects, and nephropathy (Washington Smoak et al., 2005; Joachimiak et al., 2018). Before the study, the patient II:2, a case of DBS, was initially misdiagnosed and her other clinical features were also ignored. When we found out that she suffered high myopia at a very young age and also had some other abnormal phenotypes, we realized that the patient had eoHM, which is associated with some hereditary eye diseases and is often the earliest feature of DBS. So we should pay more attention to the patient’s genetic background. For such reason, we revealed that two novel variations of LRP2 carried by the proband were followed an autosomal recessive inheritance mode and might be an early diagnostic factor for DBS patient. Besides, other animal study also showed that the LRP2-deficient eyes were first presented with increased eye lengthening by post-natal day 5 and it was accompanied by a rapid decrease of the bipolar, photoreceptor and retinal ganglion cells, and the eventually the optic nerve axons, indicating that the function of LRP2 in the ocular tissues is necessary for normal eye growth (Cases et al., 2015). Consequently, the eoHM combined with the novel LRP2 variations in our patient may provide good reference value for the diagnosis of DBS. However, the specific pathogenic mechanism by which LRP2 variations cause DBS is currently unknown and needs to be explored carefully in future studies.

## Conclusion

DBS is a complex disease with wide genetic variability and phenotypic heterogeneity. Its clinical complications are serious enough to warrant timely genetic counseling and prenatal diagnosis. The eoHM may be the first reason for children visiting ophthalmology clinic and an important clue to the clinician’s detection of underlying ocular disease. Therefore, we here identified two novel variations in *LRP2* in our DBS patient with eoHM. Further analysis in the pathogenicity of *LRP2* variations and the clinical phenotype of DBS will not only help us to understand the occurrence, development, and harmfulness of DBS but also provide a theoretical basis for the gene therapy of DBS and eugenics for patient family members in the future.

## Data Availability

The datasets for this article are not publicly available due to concerns regarding participant/patient anonymity. Requests to access the datasets should be directed to the corresponding author.

## Abbreviations

DBS, Donnai-Barrow syndrome; LRP2, low density lipoprotein receptor-related protein 2; eoHM, early-onset high myopia; BCVA, best-corrected visual acuity; AL, axial length; CC, corneal curvature; ACD, anterior chamber depth; VF, visual fields; ffERG, full-field electroretinography; OCT, optical coherence tomography; NMD, non-sense-mediated mRNA decay; M1, mutation 1: c.9032G>A (p.Arg3011Lys); M2, mutation 2: c.2909-2A>T; MRI, magnetic resonance imaging; WES, whole exome sequencing; LDL, low-density lipoprotein receptor; PTC, premature translational-termination codon.
