# Supplementary material for: Two novel variations in LRP2 cause Donnai-Barrow syndrome in a Chinese family with severe early-onset high myopia
Source: Front Genet. 2023 Jan 27;14:1107347. doi: 10.3389/fgene.2023.1107347 (PMC9911814; doi:10.3389/fgene.2023.1107347)
Supplement: Supplementary file 1 [file Table1.doc]

**Supplementary Table 1. Comparison of system features about Donnai-Barrow syndrome (DBS) between the literature and the patient presented in this paper**

| **Phenotypic manifestations of literature** | **Patient II:2** | **Reference** |
| --- | --- | --- |
| **Craniofacial anomalies** |  |  |
| broad forehead | - | Robinson et al., 2021; Avunduk et al., 2000 |
| Large anterior fontanelle | - | Chinta et al., 2011; Chassaing et al., 2003; Khalifa et al., 2015; Khan et al., 2018; Sait et al., 2021; Dachy et al., 2015 |
| Widow's peak | - | Sait et al., 2021 |
| Hypertelorism | + | Robinson et al., 2021; Chinta et al., 2011; Chassaing et al., 2003; Patel et al., 2007; Avunduk et al., 2000; Anglani et al., 2018; Shaheen et al., 2010; Roane et al., 2012; Alyousef et al., 2022; Khalifa et al., 2015; Kantarci et al., 2008; Khan et al., 2018; Sait et al., 2021; Dachy et al., 2015;,Bruce et al., 2011 |
| Prominent eyes | + | Chinta et al., 2011; Chassaing et al., 2003; Alyousef et al., 2022; Khalifa et al., 2015 |
| Downslanted palpebral fissures | - | Robinson et al., 2021; Chinta et al., 2011; Patel et al., 2007; Avunduk et al., 2000; Shaheen et al., 2010; Khalifa et al., 2015; Kantarci et al., 2008; Khan et al., 2018; Sait et al., 2021 |
| Suborbital skin creases | - | Chassaing et al., 2003; Schrauwen et al., 2014; Khalifa et al., 2015 |
| Depressed nasal bridge | + | Chinta et al., 2011; Anglani et al., 2018; Shaheen et al., 2010; Khalifa et al., 2015; Sait et al., 2021 |
| Short nose with broad tip | + | Chassaing et al., 2003; Patel et al., 2007; Anglani et al., 2018; Shaheen et al., 2010; Khalifa et al., 2015; Khan et al., 2018 |
| Cleft lip | - | Robinson et al., 2021 |
| low-set posteriorly angulated ears | - | Robinson et al., 2021; Chassaing et al., 2003; Patel et al., 2007; Avunduk et al., 2000; Higham et al., 2021; Shaheen et al., 2010; Sait et al., 2021; Bruce et al., 2011 |
| a high arched palate | - | Patel et al., 2007 |
| **Ocular features** |  |  |
| High myopia | + | Chinta et al., 2011; Schrauwen et al., 2014; Anglani et al., 2018; Shaheen et al., 2010; Khalifa et al., 2015; Kantarci et al., 2008; Khan et al., 2018; Dumitrescu et al., 2021; Storm et al., 2013; Flemming et al., 2020; Bruce et al., 2011 |
| Exotropia | - | Chinta et al., 2011; Patel et al., 2007; Avunduk et al., 2000 |
| Esotropia | + | Schrauwen et al., 2014; Khan et al., 2018 |
| Nystagmus | + | Khan et al., 2018; Dumitrescu et al., 2021 |
| amblyopia | - | Alyousef et al., 2022 |
| Persistent pupillary membrane | + | Chinta et al., 2011 |
| Iris coloboma or iris hypoplasia | - | Avunduk et al., 2000; Shaheen et al., 2010; Kantarci et al., 2008; Khan et al., 2018; Sait et al., 2021; Bruce et al., 2011 |
| Abnormal ciliary body | - | Higham et al., 2021 |
| Cataract | - | Patel et al., 2007; Schrauwen et al., 2014; Anglani et al., 2018; Kantarci et al., 2008; Khan et al., 2018 |
| Glaucoma | - | Anglani et al., 2018; Khan et al., 2018; Canut MI et al., 2020 |
| Vitreous opacities | + | Schrauwen et al., 2014; Khalifa et al., 2015 |
| Extensive chorioretinal atrophy | + | Higham et al., 2021; Khan et al., 2018; Sait et al., 2021 |
| abnomalous fovea and optic nerve | - | Chinta et al., 2011; Higham et al., 2021; Anglani et al., 2018; Kantarci et al., 2008; Khan et al., 2018; Dumitrescu et al., 2021 |
| Spontaneous retinal detachment | - | Patel et al., 2007; Schrauwen et al., 2014; Khan et al., 2018; Dumitrescu et al., 2021; Ozdemir et al., 2020 |
| **Sensorineural hearing loss** | + | Chinta et al., 2011; Chassaing et al., 2003; Patel et al., 2007; Avunduk et al., 2000; Higham et al., 2021; Anglani et al., 2018; Shaheen et al., 2010; Roane et al., 2012; Alyousef et al., 2022; Khalifa et al., 2015; Kantarci et al., 2008; Khan et al., 2018; Dumitrescu et al., 2021; Sait et al., 2021; Storm et al., 2013; Aksenova et al., 2021; Dachy et al., 2015; Bruce et al., 2011 |
| **Renal** |  |  |
| 24h Low-molecular weight^a^ proteinuria (LMWP) | 2418.95mg/24h | Chinta et al., 2011; Chassaing et al., 2003; Schrauwen et al., 2014; Anglani et al., 2018; Khalifa et al., 2015; Kantarci et al., 2008; Dumitrescu et al.,2021; Sait et al., 2021; Storm et al., 2013; Aksenova et al., 2021; Flemming et al., 2020; Dachy et al., 2015 |
| Hypercalciuria | - | Anglani et al., 2018; Aksenova et al., 2021; Flemming et al., 2020 |
| Nephrolithiasis | - | Anglani et al., 2018; |
| Focal segmental glomerulosclerosis | - | Shaheen et al., 2010; Storm et al., 2013 |
| **Congenital diaphragmatic hernia and omphalocele** | NA | Robinson et al., 2021; Chinta et al., 2011; Chassaing et al., 2003; Patel et al., 2007; Higham et al., 2021; Shaheen et al., 2010; Roane et al., 2012; Khan et al.,2018; Sait et al., 2021; Aksenova et al., 2021; Kantarci and Donahoe, 2007 |
| **Agenesis of the corpus callosum** | - | Chinta et al., 2011; Chassaing et al., 2003; Patel et al., 2007; Avunduk et al., 2000; Higham et al., 2021; Alyousef et al., 2022; Khalifa et al., 2015; Dumitrescu et al., 2021; Aksenova et al., 2021; Bruce et al., 2011 |
| **Other** |  |  |
| Joint hypermobility | **+** | Schrauwen et al., 2014 |
| Seizures | - | Kantarci et al., 2007 |
| Self-Injurious Behavior | - | Roane et al., 2012 |
| Tetralogy of Fallot | - | Robinson et al., 2021 |
| Bicornuate uterus | **+** | Longoni et al., 2018 |
| Malrotation of bowel | - | Kantarci et al., 2008; Bruce et al., 2011 |
| Scoliosis and rib/vertebral abnormalities | - | Khan et al., 2018 |

+: present, -: absent, NA: not available, 24h proteinuria: 0~124mg
